# Clinical Context Is More Important than Data Quantity to the Performance of an Artificial Intelligence-Based Early Warning System

**DOI:** 10.3390/jcm14134444

**Published:** 2025-06-23

**Authors:** Taeyong Sim, Eunyoung Cho, Jihyun Kim, Ho Gwan Kim, Soo-Jeong Kim

**Affiliations:** 1 AITRICS Corporation, Seoul 06221, Republic of Korea; ey10.cho@aitrics.com (E.C.); jhkim@aitrics.com (J.K.); 2 Department of Emergency Medicine, Presbyterian Medical Center, Jeonju 54987, Republic of Korea; hogwan2ya@hanmail.net; 3 Department of Internal Medicine, Yongin Severance Hospital, Yonsei University College of Medicine, Yongin 16995, Republic of Korea; alvin97@yuhs.ac

**Keywords:** artificial intelligence, early warning score, Charlson Comorbidity Index, electronic health records, missing data, predictive modeling

## Abstract

**Background/objectives:** The quantity of clinical data varies across patient populations and often reflect clinicians’ perceptions of risk and their decisions to perform certain laboratory tests. Missingness in electronic health records can be informative because it may indicate that certain clinical parameters were not measured because clinicians considered them unnecessary for stable patients. **Methods:** This retrospective single-center study explored the ability of a deep learning-based early warning system, the VitalCare–Major Adverse Event Score, to predict unplanned intensive care unit transfers, cardiac arrests, or death among adult inpatients 6 h in advance. We classified patients using the Charlson Comorbidity Index (CCI) and assessed whether patients with high severity and a greater volume of laboratory data benefited from more comprehensive inputs. **Results:** Overall, patients with high CCI scores underwent more testing and had fewer missing values, whereas those with moderate-to-low CCI scores underwent less testing and had more missing data. Within the event cohorts, however, the high-CCI and moderate/low-CCI groups showed similar proportions and patterns of missing values. The discriminative ability of the model remained robust across both groups, implying that the clinical context of missingness outweighed the raw quantity of available data. **Conclusions:** These findings support a nuanced view of data completeness and highlight that preserving the real-world patterns of ordering laboratory tests may enhance predictive performance.

## 1. Introduction

Clinical data are heterogeneous in real-world healthcare settings. Some patients, particularly those with complex comorbidities, undergo frequent laboratory evaluations that produce an abundance of data [1]. Other patients who are perceived as clinically stable often have sparser data available, thus raising concerns about whether machine learning models can accurately predict adverse outcomes under such incomplete circumstances. Traditionally, missing data have been viewed as problematic because missingness can lead to biases or imputed approximations [2,3]. However, previous studies have introduced the notion of “informative presence,” which suggests that the absence of laboratory tests is not random; instead, this absence signals that specific tests were not performed because no abnormality was suspected [4,5]. The VitalCare–Major Adverse Event Score (VC–MAES), which is an artificial intelligence (AI)-based early warning system designed to predict clinical deterioration events, such as unplanned intensive care unit (ICU) transfers, in-hospital cardiac arrests, or death 6 h in advance, leverages this concept by conservatively imputing missing values by assuming that the unmeasured parameters were likely within normal ranges.

Our recent study demonstrated that artificially imputing these missing values with approximate estimates reduced the performance of the VC–MAES compared with that achieved by using the system’s default normal value replacement of the system, suggesting that missing healthcare data can have intrinsic meaning and reflect the decision-making process of clinicians [6]. Furthermore, this study also demonstrated the following implicit clinical rationale: if no concern exists, then further testing may not be required. To elucidate how baseline severity intersects with these patterns of missingness, we categorized inpatients using the Charlson Comorbidity Index (CCI) to determine whether data quantity alone drives the predictive performance and whether ordering (or forgoing) laboratory tests plays a critical role in predicting outcomes relative to patients’ baseline comorbidity.

## 2. Materials and Methods

This retrospective analysis was conducted at Presbyterian Medical Center in the Republic of Korea. We included adult patients (≥19 years) admitted to the general medical–surgical wards between December 2022 and May 2024 who (i) had at least one valid measurement of each of the five key vital signs—systolic blood pressure (SBP), diastolic blood pressure (DBP), heart rate (HR), respiratory rate (RR), and body temperature—and (ii) remained hospitalized for a minimum of 24 h. Patients who were directly transferred from the emergency department or operating room to an ICU were excluded because they were considered planned ICU admissions [7,8]. Baseline comorbidities were assessed using the CCI, calculated according to methods described in previous studies [9,10]. Patients with a CCI > 3 were classified as having high severity, and those with a CCI of ≤3 were classified as having moderate-to-low severity [11].

The VC–MAES is a proprietary deep-learning model built on a bidirectional long short-term memory architecture. It outputs a risk score from 0 to 100, with higher values indicating a greater likelihood of clinical deterioration within the next 6 h. The model requires age and five core vital-sign inputs (SBP, DBP, HR, RR, and body temperature) to generate the risk score. When available, it also incorporates 13 additional physiological and laboratory variables: oxygen saturation; Glasgow Coma Scale score; and values of total bilirubin, lactate, creatinine, platelet count, pH, sodium, potassium, hematocrit, white blood cell count, bicarbonate (HCO_3_^−^), and C-reactive protein. Comprehensive specifications of the network architecture and derivation cohort were provided in our previous studies [6,12,13].

Missing values were imputed with a last-observation-carried-forward (LOCF) strategy: the most recent prior measurement replaced the missing entry. LOCF is widely utilized in clinical prediction models based on longitudinal electronic health record (EHR) data as it preserves temporal continuity and clinical plausibility by assuming relative stability between measurements. If no historical value was available, the model substituted a default normal value derived from standard reference ranges [14,15].

The composite endpoint comprised unplanned ICU transfer, in-hospital cardiac arrest, or death. The model performance was evaluated using the area under the receiver-operating characteristic curve (AUROC).

Demographic characteristics and the proportions of missing laboratory results were compared between groups classified by the CCI. Categorical variables were analyzed using the chi-square test, and continuous variables were compared using either the independent *t*-test or the Wilcoxon rank-sum test, depending on the data distribution. Differences in proportions of missingness were assessed using a two-sided z-test for independent proportions with continuity correction, based on the score method. A two-sided *p*-value of <0.05 was considered statistically significant.

## 3. Results

During this study period, 24,359 hospitalizations were recorded, including 12,139 in the high severity group (CCI > 3) and 12,220 in the moderate/low severity group (CCI ≤ 3). Patients with high severity underwent more laboratory investigations, resulting in fewer missing values and a higher rate of unplanned ICU transfer, cardiac arrest, or death (4.8%), consistent with their high risk of adverse outcomes at baseline. Conversely, patients in the moderate/low severity group underwent fewer laboratory tests and, consequently, exhibited higher missingness rates; however, they experienced significantly fewer adverse events overall (1.0%). Table 1 summarizes the baseline demographic characteristics, vital signs, and differences in laboratory test missingness of the high severity and moderate/low severity groups.

**Figure 1:**
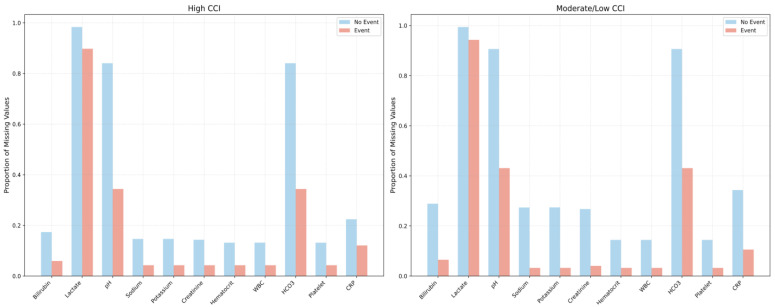
Proportion of missing laboratory test results, stratified by event vs. non-event, among patients with a high Charlson Comorbidity Index (CCI > 3) and those with a moderate/low CCI (CCI ≤ 3).

In the high-CCI and moderate/low-CCI groups, patients who experienced adverse events consistently had fewer missing laboratory values than those without events, reflecting more frequent testing when clinical deterioration was suspected. Among the event cohorts specifically, patients in both the high-CCI and moderate/low-CCI groups exhibited similar proportions of missing values overall; however, the high-CCI group had fewer missing pH and HCO_3_ values (0.34 vs. 0.43, *p* = 0.08), suggesting an even more intensive diagnostic approach for higher-risk patients (Figure 1).

**Figure 2:**
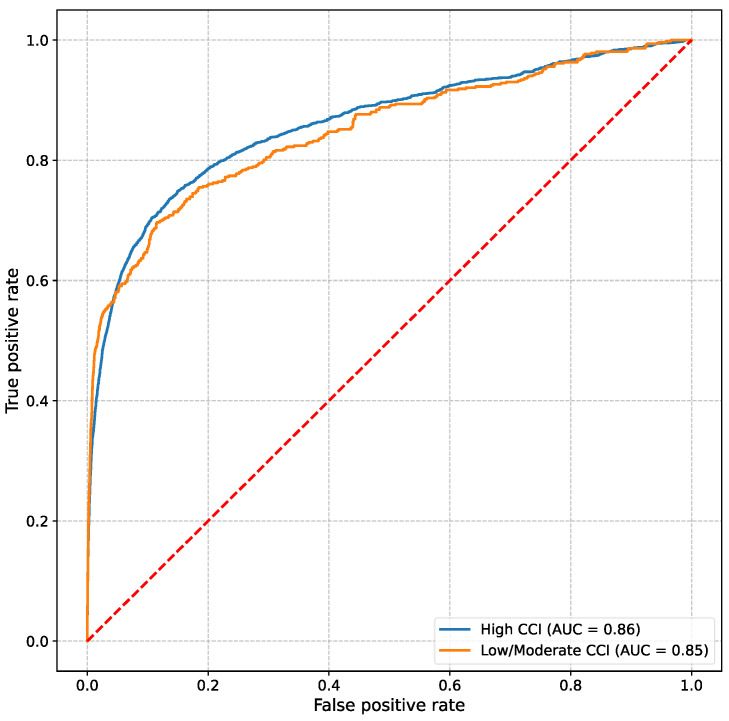
Receiver-operating characteristic (ROC) curves illustrating the areas under the ROC curve (AUC) of patients with a high Charlson Comorbidity Index (CCI > 3) and patients with a moderate/low CCI (CCI ≤ 3).

When used to predict clinical deterioration events within a 6 h prediction window, the VC–MAES achieved an AUROC of 0.86 in the overall patient cohort and maintained robust performance across both severity groups despite differences in data availability. Specifically, the AUROC values for the high severity and moderate/low severity groups were 0.86 and 0.85, respectively (Figure 2).

## 4. Discussion

Using the CCI to stratify patients into high-severity (CCI > 3) and moderate/low-severity (CCI ≤ 3) groups, we found that comorbidity burden influenced both clinical trajectories and clinicians’ propensity to order laboratory tests. Overall, patients with higher CCI scores underwent more frequent testing and therefore exhibited fewer missing values, whereas those with lower CCI scores were tested less often. However, within the event cohorts, the high-CCI and moderate/low-CCI groups displayed similar proportions and patterns of missing values, reflecting clinicians’ heightened perception of risk in these cases. The VC–MAES early-warning system retained robust discriminative performance across both strata, demonstrating that its accuracy did not depend solely on the absolute volume of laboratory data. Instead, the pattern of missingness itself served as a clinically meaningful signal—an embodiment of the “informative presence” concept, whereby each decision to order or withhold a test conveys implicit information about the clinician’s level of concern and the patient’s risk of deterioration.

Many predictive modeling approaches focus on maximizing data completeness, either by collecting more frequent measurements or by aggressively imputing missing values [16,17]. However, a recent large-scale simulation study [18] demonstrated that imputing every missing value actually deteriorates predictive performance, particularly when the same predictors are frequently missing during model deployment. Specifically, this aggressive imputation strategy leads to calibration drift and a decline in the AUROC, indicating poorer discrimination and reliability of predictions. In alignment with these findings, both our prior research and the current analysis using real-world clinical datasets highlight the importance of preserving clinically meaningful gaps. Rather than forcing data completeness by inserting synthetic values, allowing the model to retain these meaningful gaps better reflects actual clinical reasoning, thus enhancing the capability of the model to detect genuine patterns indicative of patient deterioration or clinical outcomes in practical healthcare settings [6]. This nuanced approach underscores the necessity of carefully considering both the mechanisms behind missingness and the intended deployment context when deciding how to handle missing data in predictive models.

Practically, these findings suggest that the VC–MAES can be implemented without mandating additional laboratory tests: the performance of the model depends on existing ordering patterns driven by clinical judgment, not on forcing complete data capture. Clinicians can therefore continue to order laboratory studies selectively while still receiving reliable predictions, enabling early intervention without adding cost or workflow burden.

Additionally, our findings align with those of existing literature demonstrating that incorporating not only real-world clinical practice patterns but also provider concerns can enhance the generalizability of AI-based early warning systems [19,20]. Churpek et al. compared manually collected respiratory rates documented in the EHR with automatically recorded respiratory rates measured by an FDA-approved respiratory pod device. They reported that manually collected respiratory rates differed substantially from those collected automatically. Interestingly, when using these respiratory rates to predict clinical deterioration events, such as transfers to the ICU, the manually recorded respiratory rates were significantly more accurate than those from the automated device. The authors suggested this result may indicate that manually recorded respiratory rates capture clinical information beyond physiological data alone, possibly reflecting providers’ clinical judgment or concern about patient status [21]. A recent randomized controlled trial examined the COmmunicating Narrative Concerns Entered by RNs (CONCERN) early warning system, which employs real-time patterns of nursing surveillance documentation—reflecting nurses’ concerns—as inputs to its machine-learning algorithm for predicting patient deterioration. The study demonstrated significant reductions in patient mortality, sepsis risk, and hospital length of stay with use of CONCERN. Although the VC–MAES does not directly use documentation patterns as inputs, its patterns of missingness and data collection behaviors inherently reflect clinicians’ judgments, suggesting that indirect clinical concerns could similarly enhance predictive capabilities [22].

### Limitations

This study has some limitations. First, this retrospective analysis was conducted at a single center in the Republic of Korea, inherently introducing potential biases related to local clinical practices and patient management. Local workflows and available resources could have influenced the selection and frequency of laboratory tests ordered, as well as the observed baseline comorbidity profiles. Additionally, while the CCI is a widely used measure, other severity indicators or risk stratification tools might provide alternative insights into patterns of missing data and clinical event rates. Lastly, the concept of informative presence used in this study, although valuable, inherently carries a risk of bias because it reflects clinical decision-making that can vary systematically across providers and care settings. Such decision-driven patterns of missingness may inadvertently introduce bias during model development and deployment, ultimately affecting the accuracy and generalizability of predictive outcomes. Therefore, additional multi-institutional and prospective studies are essential to comprehensively validate these findings, refine approaches for managing missing data, account for variations in testing protocols, and optimize the accuracy of risk predictions across diverse clinical settings.

## 5. Conclusions

Overall, our results indicated that respecting the natural patterns of test ordering, which often reflect clinical judgment, may be more beneficial to predictive accuracy than striving for exhaustive data. By leveraging this “informative presence”, AI-based models can balance their robustness with real-world applicability, thus ensuring that they genuinely identify patients who are at risk without necessitating unnecessary or duplicative testing. The consistent performance across CCI groups suggested that the underlying context of missingness, rather than the absolute quantity of data, plays a decisive role in model accuracy.

## Figures and Tables

**Table 1:** Baseline demographic characteristics, vital signs, and differences in missing laboratory test values for the high severity and moderate/low severity groups.

CCI Groups					
		Overall (n = 24,359)	High-CCI (n = 12,139)	Moderate/Low-CCI (n = 12,220)	*p*-Value
Age, median ± IQR, yr		69.0 ± 22.0	78.0 ± 14.0	57.0 ± 23.0	<0.001
Sex, n (%)	F	12,303 (50.5)	5456 (44.9)	6847 (56.0)	<0.001
	M	12,056 (49.5)	6683 (55.1)	5373 (44.0)	
BMI, median ± IQR, kg/m^2^		23.67 ± 5.2	22.94 ± 5.0	24.28 ± 5.1	<0.001
DBP, median ± IQR, mmHg		78.0 ± 12.0	75.0 ± 12.0	80.0 ± 15.0	<0.001
Pulse, median ± IQR		78.0 ± 18.0	79.0 ± 19.0	78.0 ± 18.0	0.006
Respiration, median ± IQR		20.0 ± 2.0	20.0 ± 2.0	20.0 ± 2.0	<0.001
SBP, median ± IQR, mmHg		125.0 ± 29.0	127.0 ± 28.0	123.0 ± 27.0	<0.001
SpO_2_ (%), median ± IQR		97.0 ± 2.0	97.0 ± 3.0	97.0 ± 2.0	<0.001
Temperature, median ± IQR, °C		36.8 ± 0.6	36.8 ± 0.5	36.8 ± 0.5	<0.001
Missing laboratory values, n (%)					
Total bilirubin		5550 (22.78)	2048 (16.87)	3502 (28.66)	<0.001
Lactate		24,038 (98.68)	11,894 (97.98)	12,144 (99.38)	<0.001
pH		20,956 (86.03)	9937 (81.86)	11019 (90.17)	<0.001
Sodium		5039 (20.69)	1723 (14.19)	3316 (27.14)	<0.001
Potassium		5045 (20.71)	1725 (14.21)	3320 (27.17)	<0.001
Creatinine		4926 (20.22)	1686 (13.89)	3240 (26.51)	<0.001
Hematocrit		3300 (13.55)	1551 (12.78)	1749 (14.31)	<0.001
White blood cell count		3303 (13.56)	1554 (12.80)	1749 (14.31)	0.001
HCO_3_^−^		20,956 (86.03)	9937 (81.86)	11,019 (90.17)	<0.001
Platelet		3300 (13.55)	1551 (12.78)	1749 (14.31)	<0.001
C-reactive protein		6832 (28.05)	2667 (21.97)	4165 (34.08)	<0.001

BMI, body mass index; CCI, Charlson Comorbidity Index; DBP, diastolic blood pressure; F, female; HCO_3_^−^, bicarbonate; IQR, interquartile range; M, male; SBP, systolic blood pressure.

## Data Availability

The data used in the current study can be obtained from the corresponding author upon reasonable request.
